# Supplementary material for: MetaRibo-Seq measures translation in microbiomes
Source: Nat Commun. 2020 Jun 29;11:3268. doi: 10.1038/s41467-020-17081-z (PMC7324362; doi:10.1038/s41467-020-17081-z)
Supplement: Supplementary file 10 — Supplementary Data 7 [file 41467_2020_17081_MOESM10_ESM.zip › File2/Confidence_VeryHigh_Taxonomy/31153_out.krona.html]

Javascript must be enabled to view this page.

members
magnitude
magnitudeUnassigned
count
unassigned
taxon
rank

31153\_out

53

2
53
superkingdom

phylum
1239
53

class
186801
53

186802
53
order

541000
53
family

40
1898205

SRS011134\_contig\_number\_contig-100\_14529.76687SRS012849\_contig\_number\_5442SRS013098\_contig\_number\_30881SRS013476\_contig\_number\_29336SRS014235\_contig\_number\_contig-100\_909.257115SRS015782\_contig\_number\_36875SRS016335\_contig\_number\_1404SRS016629\_contig\_number\_1854SRS018623\_contig\_number\_20677SRS018836\_contig\_number\_4217SRS019068\_contig\_number\_51806SRS019601\_contig\_number\_30046SRS020394\_contig\_number\_7135SRS020869\_contig\_number\_32502SRS023914\_contig\_number\_24465SRS024132\_contig\_number\_27317SRS024435\_contig\_number\_contig-100\_104.203104SRS042966\_contig\_number\_19108SRS047433\_contig\_number\_12937SRS049446\_contig\_number\_11472SRS049959\_contig\_number\_809SRS050752\_contig\_number\_19230SRS050998\_contig\_number\_15122SRS075878\_contig\_number\_17328SRS098571\_contig\_number\_contig-100\_16388.228373SRS1041032\_contig\_number\_15998SRS1041039\_contig\_number\_26229SRS1041157\_contig\_number\_1050SRS1055056\_contig\_number\_1954SRS143417\_contig\_number\_31957SRS143895\_contig\_number\_4032SRS144362\_contig\_number\_30851SRS144537\_contig\_number\_25702SRS147271\_contig\_number\_contig-100\_23632.23633SRS147346\_contig\_number\_contig-100\_17573.116772SRS148196\_contig\_number\_8999SRS149325\_contig\_number\_4880SRS149784\_contig\_number\_13422SRS893369\_contig\_number\_12325SRS971275\_contig\_number\_2891
species

13
1263
genus


SRS014412\_contig\_number\_17994SRS015431\_contig\_number\_75551SRS015578\_contig\_number\_contig-100\_12114.105039SRS015579\_contig\_number\_contig-100\_8372.8373SRS018575\_contig\_number\_3070SRS019910\_contig\_number\_contig-100\_5015.122350SRS049773\_contig\_number\_31118SRS049896\_contig\_number\_33018SRS056519\_contig\_number\_contig-100\_24580.24580SRS078242\_contig\_number\_contig-100\_38797.76418SRS892882\_contig\_number\_contig-100\_6990.78735SRS893279\_contig\_number\_contig-100\_513.89611SRS893373\_contig\_number\_contig-100\_12899.95292
species
13
1262952
